# Sporadic Creutzfeldt-Jakob Disease in 2 Plasma Product Recipients, United Kingdom 

**DOI:** 10.3201/eid2306.161884

**Published:** 2017-06

**Authors:** Patrick Urwin, Kumar Thanigaikumar, James W. Ironside, Anna Molesworth, Richard S. Knight, Patricia E. Hewitt, Charlotte Llewelyn, Jan Mackenzie, Robert G. Will

**Affiliations:** University of Edinburgh Western General Hospital, Edinburgh, Scotland, UK (P. Urwin, J.W. Ironside, A. Molesworth, R.S. Knight, J. Mackenzie, R.G. Will);; University Hospital Lewisham, London, UK (K. Thanigaikumar);; National Health Service Blood and Transplant, London (P.E. Hewitt);; National Health Service Blood and Transplant/Public Health England Epidemiology Unit, Cambridge, UK (C. Llewelyn)

**Keywords:** sporadic Creutzfeldt-Jakob disease, sCJD, variant Creutzfeldt-Jakob disease, vCJD, prions, clotting disorders, plasma products, blood transfusion, United Kingdom

## Abstract

Sporadic Creutzfeldt-Jakob disease (sCJD) has not been previously reported in patients with clotting disorders treated with fractionated plasma products. We report 2 cases of sCJD identified in the United Kingdom in patients with a history of extended treatment for clotting disorders; 1 patient had hemophilia B and the other von Willebrand disease. Both patients had been informed previously that they were at increased risk for variant CJD because of past treatment with fractionated plasma products sourced in the United Kingdom. However, both cases had clinical and investigative features suggestive of sCJD. This diagnosis was confirmed in both cases on neuropathologic and biochemical analysis of the brain. A causal link between the treatment with plasma products and the development of sCJD has not been established, and the occurrence of these cases may simply reflect a chance event in the context of systematic surveillance for CJD in large populations.

## Abstract 2

Two cases of sporadic CJD with clotting disorders have been identified, but this may represent a chance event.

Human prion diseases are a group of rare and fatal neurodegenerative diseases that include idiopathic (sporadic), genetic (inherited), and acquired (infectious) disorders (*1*). All are associated with the accumulation of an abnormal isoform of the prion protein (PrP^Sc^) in the central nervous system (*1*). The most common human prion disease is the sporadic form of Creutzfeldt-Jakob disease (sCJD), which occurs worldwide with a relatively uniform incidence of 1–2 cases per million population per year, a peak incidence in the 7th decade of life, and a median duration of illness of 4 months. The relatively consistent mortality rates associated with sCJD, the overall random spatial and temporal distribution of cases, and the absence of any confirmed environmental risk factor have led to the hypothesis that sCJD occurs because of the spontaneous generation of PrP^Sc^ in the brain (*1*). In contrast, variant Creutzfeldt-Jakob disease (vCJD) is an acquired disorder that is most likely caused by the consumption of meat or meat products contaminated with the bovine spongiform encephalopathy agent. The median age at death in vCJD is 30 years, with a median duration of illness of 14 months. Most cases of vCJD have occurred in the United Kingdom, which has had the largest epizootic of bovine spongiform encephalopathy in the world. Of the 178 UK vCJD cases, 3 have been identified as cases of secondary transmission caused by the transfusion of nonleukodepleted red blood cell components from vCJD-infected blood donors.

Lookback studies have shown no evidence of transmission through blood transfusion in sCJD (*2*,*3*), despite the identification of PrP^Sc^ in some peripheral tissues (*4*) and experimental evidence, which demonstrated infectivity in blood (*5*) by using intracerebral inoculation of highly sensitive transgenic mice. The absence of clinical cases causally linked to past treatment with fractionated plasma products has been used as evidence of the safety of these products in relation to sCJD (*6*). These products are generally manufactured from the pooled plasma from several thousand donors; production using UK plasma was discontinued in 1999.

We describe 2 cases of sCJD in patients who had previously received treatment with UK plasma–sourced plasma products; both patients had been informed that they were at increased risk for vCJD because of that treatment. The clinical features and investigations in these cases were typical of sCJD; the neuropathologic diagnosis in both cases was sCJD (subtype MM1). 

## The Investigation

The UK National CJD Research and Surveillance Unit has been carrying out systematic epidemiologic study of CJD since 1990. The methodology of this study has been published previously (*7*). In brief, patients with suspected CJD are referred by clinicians and visited by a research registrar, who obtains details of the clinical history and investigations, information on a range of possible risk factors, and past medical history. The Transfusion Medicine Epidemiology Review study investigates potential links between donors and recipients of labile blood components and, in cases of sCJD, investigates patients who have a history of blood donation or having received a blood transfusion.

Coordinated surveillance of CJD has been undertaken in the European Union since 1993 (*8*). National surveillance programs for CJD also are in place in several other countries, including Australia, Canada, Japan, and the United States.

### Case 1

In 2014, a 64-year-old woman suffered a rapidly progressive dementia with deterioration in driving skills and balance disturbance, then limb coordination deficits with handwriting impairment. In the second month, her gait deteriorated, becoming shuffling and unsteady, she struggled to dress herself, and she had onset of daytime hypersomnolence. She became distractible, had visual misperceptions, emotional lability, and spatial memory problems. She was hospitalized at the beginning of the third month of her illness and had onset of cortical blindness, myoclonus, and akinetic mutism. She experienced rapid decline and died after a total illness duration of 3 months.

An electroencephalogram performed during the final stages of illness showed background slowing and runs of periodic complexes, and a magnetic resonance imaging (MRI) brain scan showed high signal in the caudate heads with posterior cortical ribboning. A cerebral spinal fluid (CSF) 14–3–3 assay and real-time quaking-induced conversion test for PrP^Sc^ both were positive. Prion protein gene (*PRNP*) sequencing showed no mutations with methionine homozygosity at codon 129.

Postmortem examination of the brain showed widespread spongiform encephalopathy of predominantly microvacuolar type. Immunocytochemistry for prion protein gave a widespread positive reaction in a granular/synaptic pattern (Figure). No plaques or plaque-like structures were identified. Results of immunocytochemistry for disease-associated prion protein were negative in peripheral nerve, liver, lymph node, appendix, and spleen. Western blot analysis of frontal cortex and cerebellum confirmed the presence of protease-resistant prion protein with a type 1A isoform.

**Figure:**
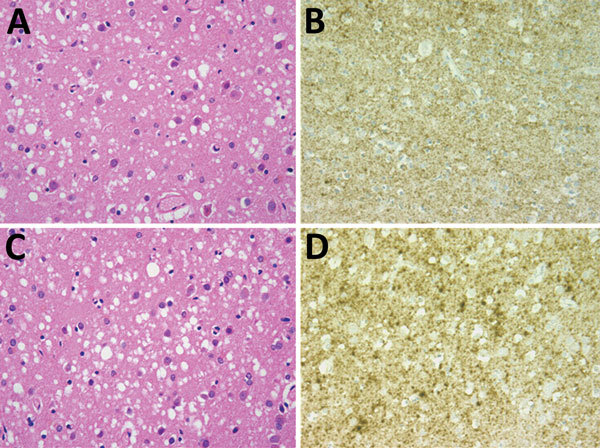
Results of neuropathologic examinations of the brains of the 2 patients with sporadic Creutzfeldt-Jakob disease, United Kingdom, 2014. A) Microvacuolar spongiform change in the frontal cortex (case 1). Hematoxylin and eosin stain; original magnification ×400. B) Fine granular/synaptic accumulation of abnormal prion protein in the cerebral cortex (case 1). 12F10 antiprion protein antibody; original magnification ×400. C) Microvacuolar spongiform change with neuronal loss and gliosis in the frontal cortex (case 2). Hematoxylin and eosin stain; original magnification ×400. D) Focally intense granular/synaptic accumulation of abnormal prion protein in the cerebral cortex (case 2). 12F10 antiprion protein antibody; original magnification ×400.

The patient had been diagnosed with von Willebrand disease in childhood. Her early therapies include numerous transfusions of red blood cells and platelets; in more recent years, she received plasma-derived and recombinant factor VIII and additional blood component transfusions at times of hemorrhage. Factor VIII was administered on 4 occasions in the 1990s and during 2000–2004 and von Willebrand factor/factor VIII (Haemate-P) during 2001–2013. Because of her history of exposure to UK-sourced plasma products, for public health purposes she had been informed that she was at risk for vCJD, although she was not known to have been exposed to factor VIII derived from a batch including a vCJD donation. She had no history of potential iatrogenic exposure to CJD and no family history of CJD.

Donors for all blood or platelet transfusions since 2001 have been identified. Of the 107 donors, 106 are still alive, with a median age of 55 years (range 27–80 years). (Table 1). One donor of leukodepleted platelets, which were transfused 12 years before clinical onset in the recipient, died in 2013 at 76 years of age, and the diagnoses on the death certificate were vascular dementia and bladder cancer. Identification of donors for transfusions before 2001 has not been possible.

**Table 1:** Selected characteristics of blood donors to the patient with sporadic Creutzfeldt-Jakob disease described in case 1, United Kingdom, 2014*

Interval from transfusion to onset, y	Component	No. donors	No. donors alive	No. donors dead
3	RBC LD	4	4	0
6	RBC LD	6	6	0
7	RBC LD	19	19	0
9	RBC LD	3	3	0
10	RBC LD	4	4	0
12	Whole blood LD; RBC LD; platelets LD	2; 27; 42	2; 27; 41	0; 0; 1


*LD, leukodepleted; RBC, red blood cells. Median age of donors, 56 years (range 27–80 years).

### Case 2

In 2014, a 64-year-old woman reported day/night reversal of sleep patterns and, 3 months later, excessive tearfulness, for which she was started on antidepressants. She then had onset of writing problems, followed during the next few days by increasing language problems that led to expressive dysphasia. She deteriorated rapidly thereafter, requiring assistance with her activities of daily living and having coordination and memory problems, jerking movements suggestive of myoclonus, and itching in both arms. She was admitted to the hospital and experienced a probable focal seizure with secondary generalization. She had onset of a homonymous hemianopia and limb rigidity and then became bedbound and mute, dying 7 months after the onset of symptoms.

An electroencephalogram performed during the final stages of illness showed widespread slowing, more evident on the left. An MRI brain scan showed left-sided caudate head and anterior putaminal high signal. Diffusion weighted imaging showed areas of cortical high signal. Results of a CSF 14–3–3 assay and real-time quaking-induced conversion tests were positive. Consent for full sequencing of the *PRNP* was not obtained; methionine homozygosity at codon 129 was identified.

Postmortem neuropathologic examination of the brain showed a widespread spongiform encephalopathy with microvacuolar spongiform change, neuronal loss, and gliosis. Immunostaining for prion protein showed widespread positivity with a granular/synaptic pattern (Figure). No amyloid plaques were identified. Western blot analysis confirmed the presence of protease resistant prion protein with a type 1A isoform. There was no evidence of abnormal prion protein accumulation in spleen and appendix either on immunocytochemistry or high sensitivity Western blot analysis.

The patient was known to have hemophilia B since 1964 and had received plasma-derived and recombinant factor IX during 1984–2012. For public health purposes, she had been informed that she was at risk for vCJD and in 1991 had received factor IX derived from a pool containing plasma from a donor who subsequently had vCJD. She had no history of potential iatrogenic exposure to CJD and no family history of CJD.

In 1985, the patient received 6 units of fresh frozen plasma (FFP). Tracing of donors has not been possible. 

## Discussion

This report describes 2 cases of sCJD in patients with a history of treatment with UK-sourced plasma products, 1 with a history of hemophilia B and 1 with von Willebrand’s disease. To our knowledge, no previous case of sCJD in a person with a history of extended exposure to plasma products has been reported. It is clearly of concern that there have been 2 such cases in a relatively short period in the UK, where many plasma product recipients have been informed that they are at increased risk for vCJD. However, a causal link between the treatment with plasma products and the onset of sCJD has not been established, and the occurrence of these cases may simply reflect a chance event in the context of systematic surveillance of CJD in large populations.

Both patients had been informed that they were at increased risk for vCJD, and considering the evidence for the type of CJD in the 2 cases is important. Both patients had a clinical phenotype suggestive of sCJD, including a short duration of illness, typical early symptoms, a suggestive MRI scan, and, in 1 patient, a typical EEG. Notably, both patients had a positive real-time quaking-induced conversion test result for PrP^Sc^ in CSF; previously this test had not been positive in any case of vCJD evaluated in our laboratory (Table 2) (*9*). However, neuropathological examination was critical; it showed appearances typical of sCJD in both patients and no evidence of peripheral pathogenesis on immunostaining of lymphoreticular tissues, a feature that is observed in all tested specimens of vCJD patients to date (*10*). Furthermore, both patients had a type 1A isoform PrP^Sc^ on Western blot consistent with a diagnosis of sCJD subtype MM1 (*11*). Neither patient had a history of potential iatrogenic exposure or a family history of CJD, and for the case for which sequencing of the *PRNP* was performed, no mutations were detected. In both cases, an MM genotype occurred at codon 129 of *PRNP*, which does not distinguish between sCJD and vCJD. Laboratory transmission studies to provide evidence of agent strain in the cases have not been possible.

**Table 2:** Selected characteristics and clinical features of the 2 patients with sporadic Creutzfeldt-Jakob disease described in cases 1 and 2, United Kingdom, 2014*

Characteristic/clinical feature	Case 1	Case 2
Patient age, y/sex	64/F	64/F
Symptoms/signs	Ataxia, cognitive impairment, visual impairment, myoclonus	Somnolence/depression, dysphasia, cognitive impairment, myoclonus/ataxia
Magnetic resonance imaging	+	+
Electroencephalogram	+	Slow activity
Cerebrospinal fluid 14–3–3 assay	+	+
RT-QuIC	+	+
Genotype	MM	MM
Diagnosis	Definite sCJD	Definite sCJD
Duration	3 mo	7 mo


*RT-QuIC, real-time quaking-induced conversion; sCJD, sporadic Creutzfeldt-Jakob disease.

One patient had received multiple transfusions of blood components over an extended period, and the other had received 6 units of FFP 19 years before clinical onset, raising the possibility that these cases could have resulted from secondary transmission through blood components. In the case of the patient with von Willebrand disease, 107 donors have been traced, and none appear in the register of cases of CJD kept at the National CJD Research and Surveillance Unit. However, it has not been possible to obtain information on blood transfusions for this patient before 2001 nor on the FFP transfusions for the patient with hemophilia B. Lookback studies in the United States and United Kingdom have provided no evidence of transfusion-transmission of sCJD (*2*,*3*), and although 1 study suggested an increase in risk after a lag period of 10 years (*12*), this finding was not confirmed in another study (*13*). The balance of evidence indicates that, if sCJD is transmitted by blood transfusion, it must be a rare event, if it happens at all, and transfusion transmission is probably not the explanation for the 2 cases we describe.

Systematic surveillance for CJD, including a coordinated study in Europe (*14*), has been carried out in many countries over the past 25 years and is continuing. Many of these studies obtain information on potential risk factors, including details of past medical history. To date, no case of sCJD has been reported in a person who has received treatment for a clotting disorder. In fact, the absence of such a case has been used to argue against the possibility that plasma-derived products pose a risk for sCJD transmission (*6*). CJD surveillance centers are aware of the relevance of this issue, and sCJD patients with a history of treatment with plasma products probably would have been identified and reported if they occurred. Although it is surprising that 2 cases of sCJD have been identified among a population of 4,000–5,000 patients in the UK who have been treated for clotting disorders with fractionated plasma products, the total population under surveillance for CJD in Europe and internationally exceeds 500 million. Assuming an annual incidence rate of sCJD of 1.5–2.0 per million population (*15*), the occurrence of 2 cases of sCJD in this total population may not imply a causal link between the treatment and the occurrence of the disease. The 2 cases were identified over a period of months, and no further cases have been found since 2014; however, continuing to search for such cases through CJD surveillance programs is essential.
